# Risk indicators of coronal and root caries in Greek middle aged adults and senior citizens

**DOI:** 10.1186/1471-2458-12-484

**Published:** 2012-06-26

**Authors:** Eleni Mamai-Homata, Vassiliki Topitsoglou, Constantine Oulis, Vasileios Margaritis, Argy Polychronopoulou

**Affiliations:** 1 Department of Preventive and Community Dentistry, Dental School, National and Kapodistrian University of Athens, 2, Thivon street, 11527, Goudi, Athens, Greece; 2 Department of Preventive Dentistry, Periodontology and Implant Biology, Dental School, Aristotle University of Thessaloniki, 54124, Thessaloniki, Greece; 3 Department of Paediatric Dentistry, Dental School, National and Kapodistrian University of Athens, 2, Thivon street, 11527, Goudi Athens, Greece

**Keywords:** Coronal caries, Root caries, Risk indicators, Middle aged adults, Senior citizens, Multivariate analysis

## Abstract

**Background:**

Dental caries is the result of a complex interplay of multiple determinants which may change overtime. Therefore, periodic surveys of caries experience and redetermination of the risk indicators of the disease are needed. The aim of this study was to assess the prevalence and severity of coronal and root caries in Greeks aged 35-44 and 65-74-year-old in relation to socio-demographic parameters. Furthermore, trends in coronal caries experience of the 35-44-year-olds were investigated.

**Methods:**

A sample of 1188 35-44-year-old and 1093 65-74-year-old individuals was selected in 2005 according to WHO guidelines for national pathfinder surveys. Caries was assessed in dentate subjects using the DMFT, DMFS, RDFS and RCI indices. Socio-demographic data were also collected. Univariate and multivariate regression analyses were performed to identify the effect of socio-demographic parameters.

**Results:**

The mean DMFT and DMFS scores of the adults were 14.06 and 45.78 respectively, while those of the senior citizens were 20.63 and 89.82. Among the 35-44-year-ods, men and those having a higher educational attainment had significantly lower DMFS values (women OR = 1.679, CI: 1.243-2.267 and >12 years of education OR = 0.321, CI: 0.193-0.535 respectively), while educational level was the only predictor of DMFS in senior citizens (OR = 0.279, CI: 0.079-0.992). The mean DMFT score of the 35-44-year-olds has not improved since 1985, but there was a remarkable reduction in the number of DT related to a simultaneous increase in the number of FT. The mean RDFS rose from 0.39 in adults to 2.66 in senior citizens. The mean RDFS score of the middle aged adults was significantly correlated with education (OR = 0.346, CI: 0.180-0.664). The RCI was almost four times greater in seniors (9.73) than in adults (2.53). There were significant differences in caries experience between the surveyed regions. MS and RDS were the major components of the DMFS and RDFS indices respectively, in both age groups.

**Conclusions:**

Caries experience in Greek adults is similar to what is observed in most industrialized countries. The mean DMFT score of the 35-44-year-olds has not improved since 1985, but a great improvement in restorative care has been observed. Senior citizens had a high percentage of untreated coronal and root surfaces. Region and education were the strongest predictors of caries experience. An increase in oral care utilization and effective prevention over the whole lifespan are needed to improve the dental health of the Greek adult population.

## Background

Dental caries is a multifactorial disease process that occurs when a susceptible tooth covered with cariogenic bacteria is frequently exposed to fermentable carbohydrates over a sufficiently long period of time. The carious process is initiated by bacterial fermentation of carbohydrates, leading to the formation of organic acids and a fall in pH, which may result in dissolution of the mineralized surface of the tooth. Besides these main factors many other endogenous and environmental factors can be introduced to either protect or further damage the tooth. Therefore, it is obvious that dental caries is the result of a very complex interplay of multiple determinants. Some of these determinants may change overtime and consequently the prevalence and severity of the disease in a population may also change. Thus, periodic surveys of the dental health status of the population and redetermination of the main risk factors and/or indicators of dental caries are needed.

By the middle of the twentieth century dental caries was well established as an endemic disease of massive proportions in most developed countries and it was the main factor responsible for dental pain and tooth loss in all ages. But during the past four decades the pattern of the disease has changed in these countries. In children, a significant decline in caries prevalence and severity has been observed [1,2], while in adults a decrease in edentulousness and an increase in remained teeth have been reported [3,4]. However, increased retention of teeth means that more adults and more tooth surfaces are at risk for caries. Therefore, dental caries, unless carefully controlled, may develop and progress throughout life. Furthermore, adults have increased risk of root caries since the prevalence of exposed root surfaces is increasing with age due to the long-term effects of trauma from inappropriate tooth brushing and gingival recession associated with periodontal disease. Thus, it is increasingly important to investigate the prevalence and severity of coronal and root caries in adults.

In Greece, the oral health of the adult population was investigated in a national oral health pathfinder survey carried out in 1985 [5]. In that survey, coronal caries experience was evaluated in persons aged 35 to 44 years. This was the only source of data at a national level, until 2005, when a second national oral health pathfinder survey was conducted. In the 2005 survey the 65-74-years-old group was also included since the aging of the population in Greece [6], as in most industrialized countries [7], and the economic, social and health consequences of this demographic evolution made the investigation of the oral health of the elderly very important. Furthermore, root caries was investigated, since it has been suggested that the aging of the population and the increasing tendency of older adults to retain their teeth will increase root caries experience and consequently the need for dental care among the elderly [8].

The main objectives of the present study were to assess the prevalence and severity of coronal and root caries in Greek individuals aged 35 to 44 and 65 to 74 years and to investigate whether dental caries experience was affected by geographical region, urbanization, gender, education and income. Furthermore, trends in coronal caries experience of the 35-44-year-olds were investigated comparing the results of the 2005 survey with those of the survey conducted in 1985.

## Methods

A sample of 1188 35-44-year-old adults and 1093 65-74-year-old senior citizens of Greek nationality was selected in 2005 according to WHO guidelines for national pathfinder surveys, which ensure the participation of a satisfactory size of people that may present different disease prevalence in the conditions that are being examined [9]. For comparison reasons the sample was collected in the same manner and from the same areas as in the survey of 1985 [5], but four new areas were also included in order to increase its size. Namely the study covered two big cities (Athens and Thessaloniki), six counties (Achaia, Chania, Evros, Ioannina, Kastoria, Larissa) and three islands (Lesbos, Naxos and Kefallinia). Three communities of different socio-economic backgrounds were selected randomly within each of the big cities, while one urban and one rural community were selected randomly within each county or island. Therefore, the survey was conducted in 24 sites (15 urban and 9 rural) and about 50 subjects were examined in each site. Samples of subjects aged 35-44 years were drawn from office and factory workers, while samples of individuals aged 65-74 years were drawn from day centers for the elderly, according to WHO national pathfinder survey methodology for these age groups [9]. Almost complete participation rate was accomplished. The Ethical Committee of the Athens Dental School gave their approval prior to the start of the study.

Prior to the survey, a meeting was organized in Athens Dental School to train and calibrate six examiners, each one of whom had the responsibility to examine the sample of individuals in four sites. Inter-examiner reliability and agreement was assessed with an experienced investigator as gold standard. For the examined indices, levels of concordance were very good (kappa coefficient > 0.85). The examinations were carried out under artificial light using dental mirrors and the WHO CPI periodontal probe. Cotton rolls and gauze were available for moisture control and removal of plaque when necessary.

The recorded variables were coronal and root caries. Coronal caries was measured using the Decayed, Missing and Filled Teeth (DMFT) and the Decayed, Missing and Filled Surfaces (DMFS) indices and was diagnosed at the caries into dentine threshold [10]. These indices are obtained by calculating the number of decayed (D), missing (M) and filled (F) teeth (T) or surfaces (S) in an individual. Root caries was recorded by the Root Decayed and Filled Surfaces (RDFS) index [9] and the Root Caries Index (RCI) [11] and was diagnosed according to WHO criteria [9]. The RDFS index is obtained by calculating the number of decayed (RDS) and filled (RFS) root surfaces, while the RCI by calculating the ratio of the number of decayed and filled root surfaces to the number of exposed root surfaces. The level of restorative care in the population examined was recorded by the Restorative Index (RI) that measures the proportion of attacked teeth which are filled (FT/FT + DT) % [12].

Socio-demographic data were collected through a structured questionnaire that was completed face-to-face at the time of the clinical examination. The participants were divided in two subgroups according to location type (urban or rural). The classification of education was based on the total number of years of education and was divided in four categories (6 years or less, 9 years, 12 years and more than 12 years). The economic status of the participants was recorded according to their monthly income and it was divided in three categories (≤590 €, 591-1760 € and ≥1761 €). Since only 4 subjects of the 65-74-year-old group belonged to the high category of income, this group was divided in two income categories (≤590 € and ≥591 €). The aforementioned classifications of the socio-demographic data were used for all the performed statistical analyses of the study.

Initially data analysis relied on descriptive statistics; data were expressed as mean ± SD or as medians ± IQR. Values of coronal caries (DMFS) were approximately normally distributed whereas for root caries (RDFS, RCI), the distribution was non-Gaussian. Examination of statistical associations was conducted with the use of *t*-test and ANOVA or Mann–Whitney test and Kruskall-Wallis test depending on the outcome's nature; parametric or non-parametric respectively. In addition, multivariate binary logistic regression analysis was performed to identify the socioeconomic risk indicators for high coronal and root caries. The cut-off point represented the last quartile of the DMFS and RDFS frequency distribution. The level of statistical significance for all tests was set at 0.05. The data were processed and analyzed by means of the statistical package for the social sciences (SPSS PC Version 19.0)

## Results

A total of 2281 subjects, 1188 adults and 1093 senior citizens were examined. Four of the adults (0.34%) and 344 of the senior citizens (31.5%) were edentulous in both jaws and were excluded from the study of dental caries. Therefore, the final sample consisted of 1184 dentate individuals aged 35-44-years and 749 dentate individuals aged 65-74 years.

### Coronal caries

Only 3 persons aged 35-44-years-old were caries free. Mean caries experience (DMFT, DMFS and components) among middle aged adults is presented in Table 1. As can be seen the mean DMFT score was 14.06, while the mean DMFS score was 45.78. Filled teeth (FT) was the major component of the DMFT index and missing surfaces (MS) was the major component of the DMFS index. Univariate analysis of the data showed that the mean DMFS score was significantly lower in men than in women (p < 0.05) as well as in those living in urban areas compared with those living in rural ones (p < 0.001). Also, the mean DMFS decreased as the educational level and monthly income of the individuals increased (p < 0.001 and p < 0.05 respectively). However, when multivariate analysis was undertaken (Table 2), only being female and having a low educational attainment remained as significant risk indicators for high DMFS score (OR = 1.679, CI: 1.243-2.267 and OR = 0.321, CI: 0.193-0.535 respectively).

**Table 1:** Mean (SD) dental caries experience (DMFT and DMFS) and Restorative Index (RI%) in 35-44-year-old Greeks by location, gender, education and monthly income

	**N**	**DT**	**MT**	**FT**	**DMFT**	**DS**	**MS**	**FS**	DMFS*	**RI %**
Location										
Rural	394	1.96	6.00	7.09	14.91 (6.03)	4.62	29.16	16.64	50.42 (27.33)	78
Urban	790	1.57	4.84	7.37	13.64 (5.70)	2.85	23.61	17.00	43.45 (23.26)	82
*t-test, p < 0.001										
Gender										
Men	611	1.85	5.09	6.65	13.47 (5.81)	3.94	24.80	15.41	44.15 (24.83)	78
Women	573	1.54	5.36	7.95	14.69 (5.81)	2.91	26.15	18.45	47.51 (24.87)	84
* t-test, p < 0.05										
Education										
≤ 6 years	131	2.68	7.81	5.19	15.51 (6.89)	6.27	37.59	12.01	55.86 (32.53)	66
9 years	99	1.91	6.98	6.01	14.79 (6.02)	4.38	33.98	14.18	52.55 (25.79)	76
12 years	396	1.93	5.53	7.49	14.78 (5.39)	3.95	27.00	17.80	48.75 (23.41)	80
> 12 years	539	1.26	4.12	7.88	13.13 (5.66)	2.22	20.12	17.94	40.28 (22.19)	86
*ANOVA, p < 0.001										
Monthly income										
≤ 590 €	127	2.24	5.91	6.72	14.65 (5.89)	5.26	28.46	15.57	49.29 (26.83)	78
591-1760 €	772	1.65	5.28	7.61	14.41 (5.67)	3.21	25.79	17.95	46.95 (23.49)	82
≥ 1761 €	64	1.28	3.72	8.31	13.16 (4.70)	2.22	18.00	19.91	40.13 (19.16)	87
*ANOVA, p < 0.05										
Total	1184	1.70	5.22	7.28	14.06 (5.84)	3.44	25.45	16.88	45.78 (24.90)	81

*s*: standard deviation.

**Table 2:** Odds ratios (OR) and 95% confidence intervals (CI) derived from multivariate binary logistic regression analysis with high coronal and root caries experience in 35-44-year-old Greeks as the dependent variable

**Dependent variable**	**Independent variables**	**OR**	**95% CI**	**p**
High DMFS^a^	Constant	0.518		0.015
	Gender (ref: male)	1.679	1.243-2.267	0.001
	Area (ref: rural)	0.787	0.571-1.085	NS
	> 12 years of education	0.321	0.193-0.535	0.0001
	≥ 1761 € monthly income	0.835	0.357-1.957	NS
High RDFS^a^	Constant	0.273		0.0001
	Gender (ref: male)	1.141	0.746-1.744	NS
	Area (ref: rural)	0.743	0.476-1.158	NS
	> 12 years of education	0.346	0.180-0.664	0.001
	≥ 1761 € monthly income	0.375	0.080-1.750	NS

^a^ The cut-off point represented the last quartile of the DMFS and RDFS frequency distribution.

*NS*: not significant.

None of the participants aged 65-74-years-old was caries free. The mean DMFT score of the senior citizens was 20.63, while the mean DMFS score was 89.82 (Table 3). The M component dominated in both indices. When univariate analysis was performed there were no significant differences in DMFS scores according to location, gender, education and monthly income in that age group. On the other hand, multiple regression analysis revealed (Table 4), that seniors with high educational level tended to have low DMFS score (OR = 0.279, CI: 0.079-0.992). The MT and MS components were more than three times higher for the senior citizens than for the adults, while the FT and FS components were almost three times higher for the adults compared to the senior citizens. The results concerning the Restorative Index (FT/FT + DT) % showed that 81% of the decayed teeth of the adults and 64% of those of the senior citizens had received treatment (Tables 1, 3).

**Table 3:** Mean (SD) coronal caries experience (DMFT and DMFS) and Restorative Index (RI %) in 65-74-year-old Greeks by location, gender, education and monthly income

	**N**	**DT**	**MT**	**FT**	**DMFT**	**DS**	**MS**	**FS**	DMFS*	**RI%**
Location										
Rural	241	1.68	15.75	2.86	20.12 (7.03)	4.59	75.27	7.16	87.02 (34.09)	63
Urban	508	1.31	17.25	2.41	20.86 (7.17)	3.07	82.07	6.01	91.16 (35.66)	65
*t-test, NS										
Gender										
Men	427	1.67	17.03	2.06	20.63 (7.49)	4.21	80.98	5.15	90.34 (36.87)	55
Women	322	1.12	16.42	3.21	20.62 (6.63)	2.71	78.43	8.01	89.14 (32.87)	74
*t- test, NS										
Education										
≤ 6 years	586	1.54	17.22	2.18	20.82 (7.33)	3.91	81.91	5.29	91.11 (36)	59
9 years	59	1.58	16.20	3.36	21.03 (6.01)	3.59	77.49	8.8	89.88 (30.83)	68
12 years	68	0.76	14.84	3.94	19.37 (6.82)	1.72	71.44	10.99	84.15 (31.61)	84
> 12 years	31	0.61	13.26	5.06	18.90 (5.19)	1.45	64.10	12.29	77.84 (31.23)	89
*ANOVA, NS										
Monthly income										
0-590 €	384	1.32	17.04	2.47	20.70 (7.21)	3.43	81.08	6.38	90.89 (35.49)	65
≥ 591 €	126	1.07	16.99	2.91	20.85 (6.75)	2.42	81.11	7.29	90.83 (34.53)	73
*t-test, NS										
Total	749	1.43	17.76	2.55	20.63 (7.12)	3.56	79.88	6.38	89.82 (35.19)	64

*NS*: not significant.

*SD*: standard deviation.

**Table 4:** Odds ratios (OR) and 95% confidence intervals (CI) derived from multivariate binary logistic regression analysis with high coronal and root caries experience in 65-74 year-old Greeks as the dependent variable

**Dependent variable**	**Independent variables**	**OR**	**95% CI**	**p**
High DMFS^a^	Constant	0.315		0.0001
	Gender (ref: male)	0.824	0.535-1.270	NS
	Area (ref: rural)	1.437	0.910-2.267	NS
	> 12 years of education	0.279	0.079-0.992	0.049
	≥ 591 € monthly income	1.123	0.669-1.887	NS
High RDFS^a^	Constant	0.896		0.541
	Gender (ref: male)	0.691	0.469-1.019	NS
	Area (ref: rural)	0.801	0.541-1.184	NS
	> 12 years of education	0.429	0.152-1.211	NS
	≥ 591 € monthly income	0.688	0.425-1.113	NS

^a^ The cut-off point represented the last quartile of the DMFS and RDFS frequency distribution.

*NS*: not significant.

**Figure 1:**
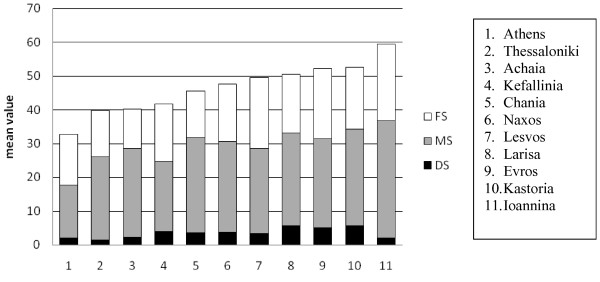
Dental caries experience (mean DMFS and components) among 35-44-year-olds in the surveyed regions of Greece in 2005.

Figure 1 presents the regional differences in DMFS values for the 35-44-year-olds. The lowest score (32.78) was found for subjects living in Athens (urban population) and the highest (59.54) for individuals living in Ioannina County (urban and rural population). There were significant differences in caries experience between the surveyed areas (p < 0.001). The regional differences in DMFS index for the 65-74-year-olds were not significant and ranged between 81.22 in Chania County and 98.90 in Larissa County (data not shown).

### Root caries

Root caries affected 11.1% of the 35-44-year-olds and 38.3% of the 65-74-year-olds (Tables 5, 6). The mean number of decayed and filled root surfaces (RDFS) rose from 0.39 in adults to 2.66 in senior citizens. Decayed root surfaces (RDS) were the major component of the RDFS index in both age groups. Univariate non-parametric tests showed that the mean RDFS score of the adults was significantly lower in those living in urban areas compared to those living in rural ones (p < 0.001) and decreased significantly as their educational level increased (p < 0.01). The mean RDFS of the seniors was significantly lower in women than in men (p < 0.05) and decreased significantly as their educational level and monthly income increased (p < 0.05). Furthermore, significant differences (p < 0.001) in RDFS values were observed between the surveyed areas in both age groups (data not shown). Multiple regression analysis (Table 2) revealed that only adults 35-44-years-old with high educational attainment were less probable to have high RDFS score (OR = 0.346, CI: 0.180-0.664).

**Table 5:** Mean root caries experience (RDFS and RCI) in 35-44-year-old Greeks by location, gender, education and monthly income (first line: mean and standard deviation in parenthesis, second line: median and interquartile range in parenthesis)

	**N**	**RDS**	**RFS**	RDFS*	**% subjects with R-DFS**	**n**	**RCI%**
Location							
Rural	394	0.62	0.11	0.73 (3.20)	15.3	269	3.13 (11.6)
				0.00 (0.00)			0.00 (0.00)
Urban	790	0.14	0.07	0.21 (1.10)	8.9	398	2.14 (8.30)
				0.00 (0.00)			0.00 (0.00)
*Mann–Whitney U test, p < 0.001							
Gender							
Men	611	0.32	0.07	0.39 (2.23)	11.1	336	2.90 (10.7)
				0.00 (0.00)			0.00 (0.00)
Women	573	0.28	0.10	0.38 (1.89)	11.0	331	2.30 (8.70)
				0.00 (0.00)			0.00 (0.00)
*Mann–Whitney U test, NS							
Education							
≤ 6 years	131	0.56	0.07	0.63 (2.02)	10.7	79	3.72 (12.34)
				0.00 (0.00)			0.00 (2.72)
9 years	99	0.4	0.05	0.45 (1.50)	16.2	59	2.24 (6.45)
				0.00 (0.00)			0.00 (0.00)
12 years	396	0.35	0.10	0.45 (2.20)	11.1	215	3.06 (10.7)
				0.00 (0.00)			0.00 (0.00)
> 12 years	539	0.13	0.07	0.20 (1.50)	8.2	304	1.79 (8.68)
				0.00 (0.00)			0.00 (0.00)
*Kruskal-Wallis test, p < 0.01							
Monthly income							
≤ 590 €	127	0.31	0.05	0.36 (1,36)	13.4	62	1.89 (6.30)
				0.00 (0.00)			0.00 (1.06)
591-1760 €	772	0.17	0.10	0.27 (1,22)	13.5	434	2.57 (10.0)
				0.00 (0.00)			0.00 (0.00)
≥ 1761 €	64	0.00	0.11	0.11 (0,67)	3.1	38	0.31(1.35)
				0.00 (0.00)			0.00 (0.00)
*Kruskal- Wallis test, NS							
Total	1184	0.3	0.09	0.39 (2.00)	11.1	667	2.53 (10.0)
				0.00 (0.00)			0.00 (0.00)

*NS*: not significant.

*n*: number of subjects with exposed root surfaces.

**Table 6:** Mean root caries experience (RDFS and RCI) in 65-74-year-old Greeks by location, gender, education and monthly income (first line: mean and standard deviation in parenthesis, second line: median and interquartile range in parenthesis)

	**N**	**RDS**	**RFS**	RDFS*	**% subjects with R-DFS**	**n**	**RCI%**
Location							
Rural	241	3.00	0.12	3.11 (7.10)	41.5	219	9.64 (17.80)
				0.00 (3.00)			0.00 (12.95)
Urban	508	2.22	0.22	2.45 (5.90)	24.6	405	9.79 (18.00)
				0.00 (2.00)			0.00 (7.10)
*Mann–Whitney U test, NS							
Gender							
Men	427	3.06	0.18	3.24 (7.10)	40.5	356	11.14 (19.7)
				0.00 (3.00)			0.00 (11.2)
Women	322	1.69	0.20	1.89 (4.90)	35.4	268	7.88 (15.2)
				0.00 (1.00)			0.00 (6.00)
*Mann–Whitney U test, p < 0.05							
Education							
≤ 6 years	586	2.75	0.14	2.89 (6.60)	40.3	489	10.24 (18.2)
				0.00 (2.00)			0.00 (11.1)
9 years	59	2.27	0.49	2.76 (6.40)	40.7	51	9.52 (16.7)
				0.00 (2.00)			0.00 (3.60)
12 years	68	1.15	0.39	1.54 (3.40)	30.9	56	8.5 (19.11)
				0.00 (1.25)			0.00 (5.90)
> 12 years	31	1.06	0.10	1.16 (4.70)	19.4	24	2.42 (7.30)
				0.00 (0.00)			0.00 (0.43)
*Kruskal-Wallis test, p < 0.05							
Monthly income							
≤ 590 €	384	2.39	0.15	2.54 (6.09)	39.3	320	9.23 (18.3)
				0.00 (2.00)			0.00 (9.60)
≥591€	126	1.63	0.17	1.80 (5.65)	27.8	100	6.60 (14.2)
				0.00 (1.00)			0.00 (5.00)
*Mann–Whitney U test, p < 0.05							
Total	749	2.48	0.19	2.66 (6.30)	38.3	624	9.73 (18.0)
				0.00 (2.00)			0.00 (11.51)

*NS*: not significant.

*n*: number of subjects with exposed root surfaces.

The Root Caries Index (RCI) was almost four times greater in seniors (9.73) than in adults (2.53). In both age groups, no significant differences in RCI scores by location, gender, education and monthly income were found. On the other hand, RCI showed significant variation by region (p < 0.001) (data not shown).

## Discussion

The main objective of this study was to provide data on coronal and root caries experience of 35-44 and 65-74-year-old Greeks in relation to socio-demographic parameters. Furthermore, trends in coronal caries experience of the 35-44-year-olds were investigated comparing the results of the present survey with those of a survey conducted in 1985. For this reason the sample was collected in the same manner and from the same areas as in 1985, but four new areas were also included. Although the sample cannot be characterized as random, it can be considered as illustrative of the whole population since it ensures the participation of a satisfactory size of people leaving in representative urban and rural areas of Greece.

According to the results of the study, the level of coronal caries experience in Greek middle-aged adults and senior citizens seems to be similar or lower to what is observed in most European countries [4,13-16] the United States [17] and Canada [18]. However, the D and M components are relatively higher and the F component relatively lower than those reported for some of these countries [13-15]. These findings may be attributed to inadequate utilization of dental services, since in a recent study it was found that a low percentage of Greek adults (39.6%) reported having visited a dentist within last year, compared to the average (62%) of the EU-25 countries [19]. They may also reflect higher supply of and demand for radical treatment and less positive attitudes towards dental health.

The comparison of the results between the two age groups showed that similar to other studies [4,15,16,20] caries experience varied by age and it was greater in senior citizens mainly because of the high number of missing teeth. It also showed that the mean number of filled teeth was much greater in adults than in senior citizens, and therefore the adults had a higher Restorative index (RI). However, 19% of the decayed teeth of the adults and 36% of those of the senior citizens remained untreated. The high percentage of untreated teeth in individuals aged 65-74-years-old may be due to difficulties in accessing dental services and lack of interest for dental health.

The finding of the multivariate analysis that the mean DMFS values of the 35-44-year-olds were significantly lower in men and in those having a higher educational attainment supports those of previous studies [15,21,22]. Significant differences in DMFS scores according to education were also found in the 65-74-years-olds similarly to other studies [15,21].

**Figure 2:**
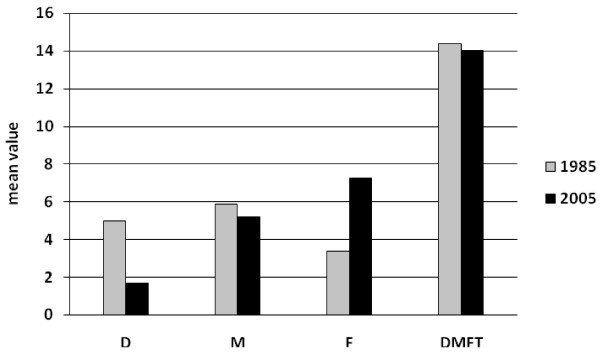
Mean DT, MT, FT and DMFT values of 35-44-year-old Greeks in 1985 and 2005.

Comparisons of the present findings with those of 1985, that have not been published but are stored in the WHO Oral Health Data Bank [23], indicate that there were no changes in DMFT scores of the middle aged adults over the 20 year period between the surveys (Figure 2). These findings are in accordance with those observed in other countries [16,20], although in some, a decline in caries experience has been reported [4,15]. This decline is mainly attributed to a reduction in the number of missing teeth. In our study the MT component was slightly lower in 2005 (5.22) compared to that in 1985 (5.90) and may be this is the reason for the slight reduction in DMFT score. The mean value of decayed teeth fell considerably as compared with the 1985 study, from 4.80 to 1.70, while the mean value of filled teeth increased from 4.00 to 7.28. However, it must be considered that in 1985 the examination for dental caries was conducted with a sharp explorer [24], whereas in 2005 the Community Periodontal Index probe was used to confirm visual evidence of caries [9]. Therefore, an underestimation of the carious lesions in the 2005 survey is probable. The restorative index increased from 45% in 1985 [5] to 81% in 2005, indicating a remarkable improvement in restorative care.

Since in the survey of 1985 subjects aged 65-74-years-old had not been examined, there are not comparable data in a national level for this age group. However, there are epidemiological data for two of the areas examined in the present study, Athens and Thessaloniki. According to these data, the mean DMFT value in subjects living in Athens in 1995-96 was 23.9 [25] and in those living in Thessaloniki in 1998-99 was 20.26 [26]. The corresponding mean values in our study were 20.39 in Athens and 21.10 in Thessaloniki. So, it seems that a reduction in dental caries experience of the elderly has taken place in Athens and a slight increase of the DMFT score has taken place in Thessaloniki. However, it must be considered that these studies were performed by different examiners under different field conditions and therefore the possibility of variations in clinical measurements cannot be excluded.

Similar to other studies [21,27-29] the present study showed that root caries varied by age and it was more frequent in the senior citizens. The low proportion of RFS in RDFS in both age groups showed that most of the root caries lesions were not restored, but as in coronal caries, the number of untreated lesions was much greater in the older age group, suggesting lower utilization of dental health services by the elderly. The finding that the mean RDFS scores of the middle aged adults were associated with educational level supports the view that education is a strong indicator of caries experience.

Comparisons of the root caries findings with those of other studies should be done with caution, since there is a great variation of results in the literature. This discrepancy may be due to differences in geographic location, population characteristics, diagnostic criteria and sampling procedures. It may also be attributed to the fact that root caries experience depends greatly on the number of retained teeth as well as on the number of exposed root surfaces that differ in the various studies. However, it seems that root caries experience in the subjects of the present study was similar or lower to that estimated for populations of most other countries [27,28,30-32]. But contrary to some of these studies [28,30,31], untreated caries on root surfaces (RDS) accounted for the major part of the mean root caries experience (RDFS).

## Conclusions

Coronal and root caries experience in Greek middle-aged adults and senior citizens is similar to what is observed in most industrialized countries. The mean DMFT score of the 35-44-year-olds has not improved since 1985, indicating the need for effective prevention over the whole lifespan. Although, there was a remarkable reduction in the number of decayed teeth of middle-aged adults in 2005, related to a simultaneous increase in the number of filled teeth, there is room for further improvement. Socio-demographic variables and especially region and education significantly affected caries experience. The great number of untreated carious lesions of the elderly indicates that an improvement in oral health services is needed to cover more efficiently their dental needs.

## Competing interests

The authors declare that they have no competing interests

## Authors’ contributions

EM-H: co-organised the epidemiological survey, made the analysis and interpretation of the data and drafted the manuscript. VT: co-organised the epidemiological survey and participated in drafting the paper. CO: conceived the study and co-organised the epidemiological survey. VM: performed the statistical analysis of the data. AP: contributed in the design of the study and checked the statistical analysis. All authors read and approved the final manuscript.

## Pre-publication history

The pre-publication history for this paper can be accessed here:

http://www.biomedcentral.com/1471-2458/12/484/prepub
